# Oligonucleotide therapeutics for neurodegenerative diseases

**DOI:** 10.1515/nipt-2024-0013

**Published:** 2024-11-18

**Authors:** Victor Li, Yunlong Huang

**Affiliations:** University High School, Irvine, CA, USA; Ting Therapeutics, Inc., San Diego, CA, USA

**Keywords:** oligonucleotide therapeutics, Alzheimer’s disease, amyotrophic lateral sclerosis, antisense oligonucleotides, small interfering RNA

## Abstract

Recently there has been a surge in interest involving the application of oligonucleotides, including small interfering RNA (siRNA) and antisense oligonucleotides (ASOs), for the treatment of chronic diseases that have few available therapeutic options. This emerging class of drugs primarily operates by selectively suppressing target genes through antisense and/or RNA interference mechanisms. While various commercial medications exist for delivering oligonucleotides to the hepatic tissue, achieving effective delivery to extra hepatic tissues remains a formidable challenge. Here, we review recent advances in oligonucleotide technologies, including nanoparticle delivery, local administration, and 2′-O-hexadecyl (C16)-conjugation that work to extend the applicability of siRNAs and ASOs to nerve tissues. We discuss critical factors pivotal for the successful clinical translations of these modified or engineered oligonucleotides in the context of treating neurodegenerative diseases such as Alzheimer’s disease and amyotrophic lateral sclerosis.

## Introduction

Oligonucleotide-based medications have the capacity to suppress the production of disease-associated proteins by interfering at the transcript level. This sets them apart from antibody-based treatments that target disease proteins either secreted or expressed on the cell surface. Therefore, oligonucleotides are poised to emerge as a distinct category of drugs, complementing small molecules and biologics, all of which play crucial roles in the advancement of modern medicine. Notable examples of oligonucleotide-based therapeutics include small interfering RNA (siRNA) and antisense oligonucleotide (ASO). The siRNAs are synthetic double-stranded RNA molecules that function by targeting and dismantling disease-causing mRNA through RNA-induced silencing complex (RISC) mediated RNA interference (RNAi). In contrast, ASOs are primarily single-stranded short fragments of DNA that bind complementary mRNA and cause sequence-specific cleavage of the RNA by endonuclease RNase H.

**Figure 1:**
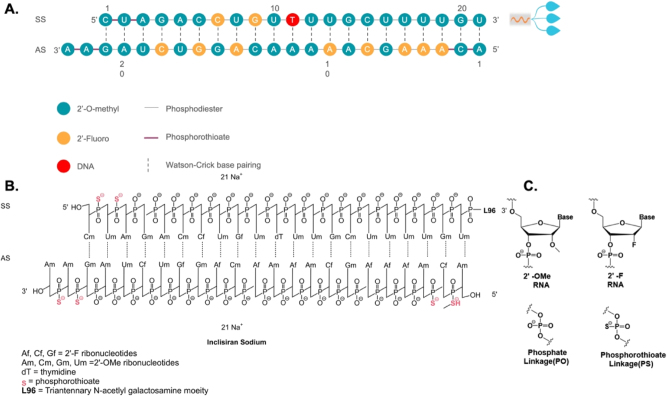
Sequence and chemical structure of GalNAc-conjugated Inclisiran. A Sequence of Inclisiran. B Chemical structure of Inclisiran with chemical modifications of oligonucleotides, conjugated with L96 triantennary GalNAc (N-acetylgalactosamine) residues to enable hepatocyte-targeted delivery. C Chemical structure of modified oligonucleotides. SS, sense strand; AS, antisense strand.

The siRNA and ASO drugs are commonly denoted by the suffixes “siran” and “-rsen”, respectively. Figure 1 illustrates a representative siRNA drug, Inclisiran. Inclisiran is considered a first-in-class siRNA drug by the Food and Drug Administration (FDA) for heterozygous familial hypercholesterolemia, a genetic condition that causes severely high cholesterol, and clinical atherosclerotic cardiovascular disease, which involves cholesterol buildup in the arteries [1]. A siRNA drug typically consists of oligonucleotides with a length ranging from 19 to 23 nucleotides. Inclisiran has an antisense strand composed of 23 nucleotides and a sense strand of 21 nucleotides. The antisense strand overhangs the sense strand, which serves as a passenger to allow the interaction with RISC and subsequent RNAi (Figure 1A). Oligonucleotide molecules are hydrophilic and large in size. They are highly polar molecules that do not successfully traverse cellular membranes by passive diffusion. Therefore, their cellular uptake remains challenging. Inclisiran is unique in that its 3′ end of the sense strand is linked to triantennary N-acetylgalactosamine (GalNAc), which facilitates a direct interaction with asialoglycoprotein to enter hepatocytes (Figure 1A). Within hepatocytes, the antisense strand of Inclisiran binds complementarily with target RNA and leads to its knockdown through the endogenous RNAi pathway [2]. Since the target RNA is proprotein convertase subtilisin/kexin type 9 (PCSK9), a key protein in the low-density lipoprotein cholesterol (LDL-C) metabolic pathway, Inclisiran decreases levels of LDL-C, thereby providing cardiovascular benefits.

## Chemical modifications of siRNA

RNAi through siRNA holds promise for treating chronic diseases with unmet medical needs. However, there are several obstacles to the clinical application of siRNA. First, unmodified siRNAs have a short half-life and poor stability in the body. Free RNA that enters the bloodstream is quickly filtered and removed by the kidneys. Furthermore, ribonucleases, which are prevalent in human blood and tissue fluid, are specialized enzymes dedicated to the breakdown of circulating siRNAs. Second, the siRNA molecules are too large and hydrophilic to passively pass through cell membranes for cytosolic release, where they exert their biological functions. Third, exogenous nucleic acid molecules are immunogenic and can easily trigger an undesirable immune response in the body. These systemic obstacles suggest that unmodified siRNAs cannot be utilized directly as medication without certain chemical modifications. Chemical modification of siRNA represents an efficient approach to overcome these issues and enhance the drug-like qualities of siRNA. Significant progress has been achieved in chemically or structurally altering specific functional groups while preserving the fundamental chemical structure of the drug. Through such modifications, improvements can be made not only in the stability and effectiveness of siRNA drugs but also in mitigating adverse effects. These chemical alterations encompass modifications of nucleotide’s ribosugar moiety with 2′-deoxy-2′-fluoro (2′-F) or 2′-O-methyl (2′-OMe), as well as modifications of the phosphorothioate (PS) backbone to replace the original phosphodiester bond, thereby enhancing the drug-like qualities and chemical stability (Figure 1B and C). Additionally, the incorporation of metabolically stable 5′-(E)-vinylphosphonate at the 5′ end of the antisense strand significantly enhances the *in vitro* potency and stability of siRNA. Moreover, the inclusion of glycol nucleic acid (GNA) within the siRNA’s antisense strand’s seed region enhances thermal and metabolic stability, reduces off-target toxicity, and maintains on-target activity.

## Organ-specific delivery of oligonucleotide

### Hepatic GalNAc delivery system

One of the most significant breakthroughs in tissue-specific oligonucleotide therapies is the hepatic delivery of siRNA via the GalNAc delivery system. Clinical application of Inclisiran and Zilebesiran using GalNAc as a hepatic delivery system are administered subcutaneously and have been shown to lower LDL cholesterol levels and prevent atherosclerotic cardiovascular disease (ASCVD) [3]. GalNAc conjugated with siRNA enables targeted delivery of siRNAs to hepatocytes by directly conjugating siRNAs to a triantennary GalNAc sugar [4]. This approach allows for receptor-mediated endocytosis via the asialoglyco protein receptor (ASGPR), which is predominantly expressed on the surface of hepatocytes. Upon uptake, a fraction of GalNAc-siRNA molecules reaches the cytoplasm and loads into the Argonaute protein in the RISC. Subsequently, the sense strand of the siRNA is released, and the antisense strand facilitates sequence-specific enzymatic cleavage by guiding RISC to the complementary RNA, thereby decreasing protein expression in a highly targeted manner. While therapeutics based on siRNA delivered to hepatocytes have been approved, new delivery solutions are needed to target additional organs.

### Brain-specific 2′-O-hexadecyl (C16) delivery system

Delivery of siRNA to extrahepatic tissues, such as the central nervous system (CNS), is exceedingly difficult. Recently, conjugation of 2′-O-hexadecyl (C16) to siRNAs has been demonstrated to target broad cell types beyond liver tissue and exert safe, strong, and long-lasting silencing in the lung, eye, and CNS of rodents and non-human primates [5]. Further improvements by administering C16-siRNAs intrathecally or intracerebroventricularly have resulted in active siRNA delivery across CNS areas and cell types, as well as sustained RNAi activity. This intracerebroventricular delivery of siRNA has been evaluated in a preclinical mouse model of Alzheimer’s disease, which showed that this amyloid precursor protein-targeting siRNA improved behavioral and physiological abnormalities [5]. These data suggest that C16 conjugation of siRNAs offers promising potential in safely and therapeutically silencing target genes outside of the liver.

**Figure 2:**
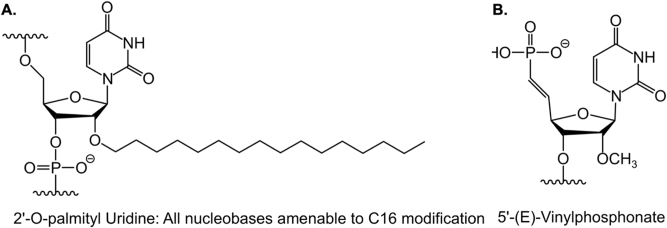
Chemical structures of C16 and 5′-(E)-VP. A Chemical structure of 2′-O-hexadecyl (C16) for central nervous system delivery. B Chemical structure of 5′-(E)-vinylphosphonate (VP), a lipophilic moiety to enhance central nervous system delivery.

Another approach for the brain-specific delivery of siRNAs and ASOs involves the use of lipophilic moieties. This method has been demonstrated to enhance cellular absorption and facilitate the delivery of siRNAs and ASOs to lipid-rich organs such as the brain. Moreover, the intracellular transport of siRNA and ASOs can be enhanced without compromising their broad biodistribution, potency, or safety by carefully adjusting their lipophilicity. Since the 2′ position of the ribose sugar backbone provides numerous options for the placement of the lipophilic moiety within the siRNA duplex, it is commonly utilized for inserting the lipophilic moiety. This approach, combined with established siRNA design components such as the deliberate insertion of 2′-fluoro and 2′-O-methyl chemical modifications to enhance potency or glycol nucleic acid (GNA) in the antisense seed area to improve specificity, offers significant advantages in terms of CNS delivery compared to other approaches. For instance, when evaluated side-by-side, C16 and/or 5′-(E)-vinylphosphonate (VP), a 5′ phosphate mimic positioned at the antisense strand’s 5′ end to facilitate RISC loading (Figure 2), emerge as the most effective siRNA across all CNS regions, achieving knockdown rates of up to 90 % of the targeted mRNA.

### Lipid nanoparticle (LNP) delivery system

**Figure 3:**
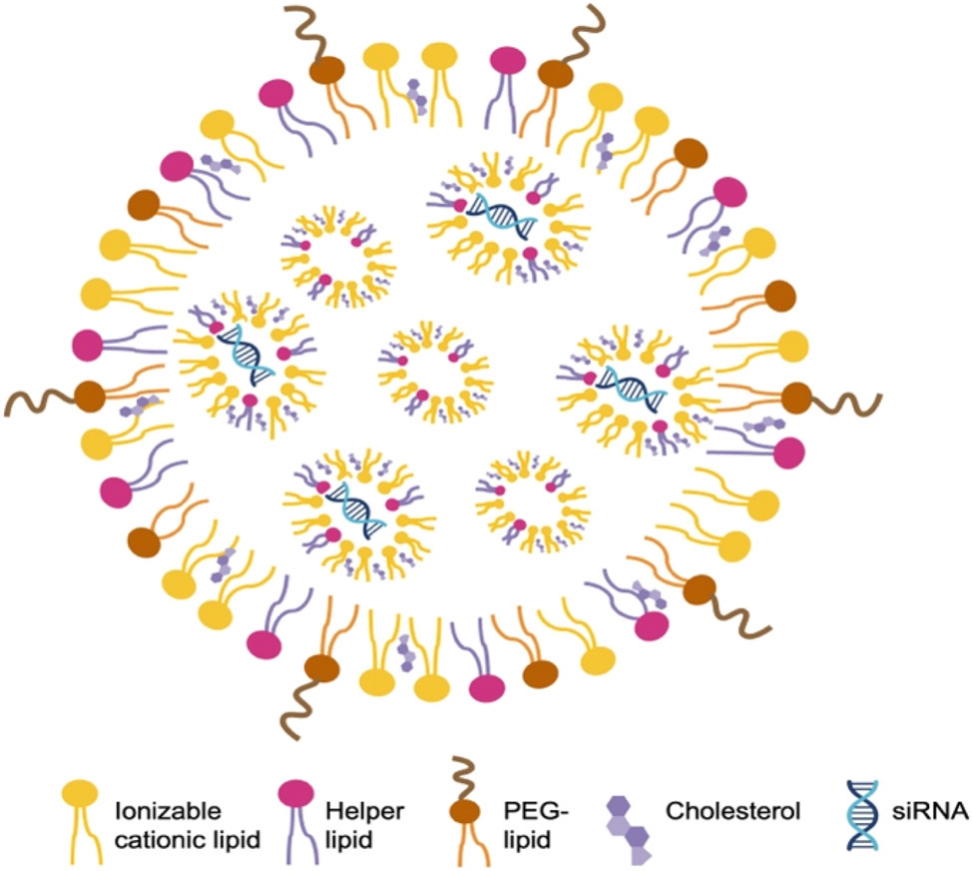
Representative composition and structure of LNPs containing siRNA phospholipids, cholesterol, ionizable cationic lipids, and PEG-lipids are typical constituents of LNPs. These molecules have hydrophilic heads and hydrophobic tails that enable self-assembly into membrane structures. The ionizable cationic lipids are positively charged, allowing close interaction with negatively charged siRNA, which forms the basic structure of LNPs.

The siRNAs are initially delivered using liposomes to preserve the stability of the siRNA. Liposome-based siRNA delivery has shown promising pharmacological effects in animal studies. However, liposomes are not very stable in blood and also have considerable toxicities *in vivo* [6]. Fortunately, significant progress has been made to improve the encapsulation of siRNAs. Cationic lipid-based LNPs and later ionizable lipid-based LNPs were developed and soon showed success in RNAi delivery to different target organs. In these LNPS, phospholipids, cholesterol, ionizable cationic lipids, and polyethyleneglycol (PEG)-lipids were rigorously screened and identified as the main constituents (Figure 3). These molecules have hydrophilic heads and hydrophobic tails that facilitate the formation of spherical membrane structures. Within these structures, the negatively charged siRNA interacts with the positively charged ionizable cationic lipids, forming the basic structure of LNPs. Lipids other than ionizable cationic lipids also serve specific functions in LNPs. For example, phospholipids act as helper lipids, cholesterol enhances cell entry, and PEG maintains stability and prevents serum protein binding [7], [8], [9]. Rational design techniques were combined with iterative screening procedures to determine the optimal configuration and makeup of alkyl chains, linkers, and amino head groups. In 2018, the FDA approved the use of patisiran (Onpattro), a first-in-class medication formulated into LNPs, to treat polyneuropathy [10]. siRNA therapeutics utilizing LNP delivery are typically administered intravenously. The LNPs protect the siRNA and facilitate its delivery to the liver to reduce the abnormal form of transthyretin, which is the pathogenic factor in polyneuropathy.

**Figure 4:**
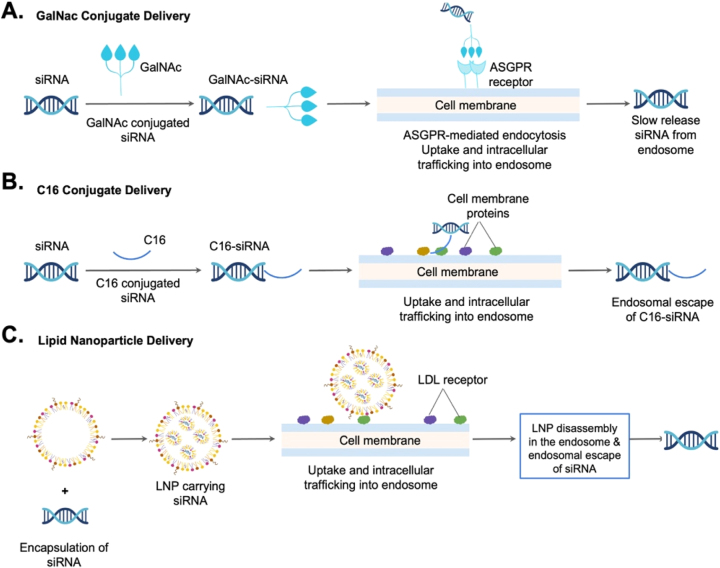
Comparison of three siRNA targeting delivery systems: A hepatic delivery system employs GalNAc directly conjugated to siRNA. B Extrahepatic tissue delivery system, such as the central nervous system (CNS) delivery system, employs C16 directly conjugated to siRNA. C Lipid nanoparticle (LNP) for extrahepatic delivery system employs a ligand or antibody conjugated to a nanoparticle to target a receptor expressed on the cell.

Delivering oligonucleotides to specific tissues remains challenging. Here, we summarize three siRNA targeting delivery systems. First, in the hepatic delivery system, GalNAc is directly conjugated to siRNA to facilitate target cell recognition (Figure 4A). Next, in the CNS delivery system, C16 is directly conjugated to siRNA to help pass the BBB and reach brain cells (Figure 4B). Finally, in the LNP delivery system, a ligand or antibody is conjugated to a nanoparticle to target a receptor expressed on the specific cell type (Figure 4C). These novel siRNA targeting delivery systems offer a variety of tissue specificity and continue to thrive in the current biotechnological boom.

## RNAi therapeutics to treat neurodegenerative diseases

In addition to treating metabolic diseases, RNAi drugs offer a promising avenue for addressing several CNS diseases that currently have unmet medical needs, such as Alzheimer’s disease, ALS, frontotemporal dementia, cerebral amyloid angiopathy, Huntington’s disease, multi-system atrophy, Parkinson’s disease, and spinocerebellar ataxia. The most prevalent age-related neurodegenerative illness is Alzheimer’s disease (AD), which is defined by a steady decline in cognitive function [11]. Over 50 million people worldwide were impacted by AD in 2019, and by 2050, that number is predicted to rise to 152 million. Furthermore, the $1 trillion annual cost of AD globally is expected to quadruple by 2030. Currently, there is no disease-modifying therapy for AD. Palliative treatment options available at this time are clinical therapy with acetylcholinesterase inhibitors or N-methyl-d-aspartate receptor antagonists, which offer limited improvement in cognition and behavior for Alzheimer’s patients and do not slow the disease’s progression [12]. Therefore, there is a critical need to develop treatments that target the pathogenic pathways underlying AD.

**Figure 5:**
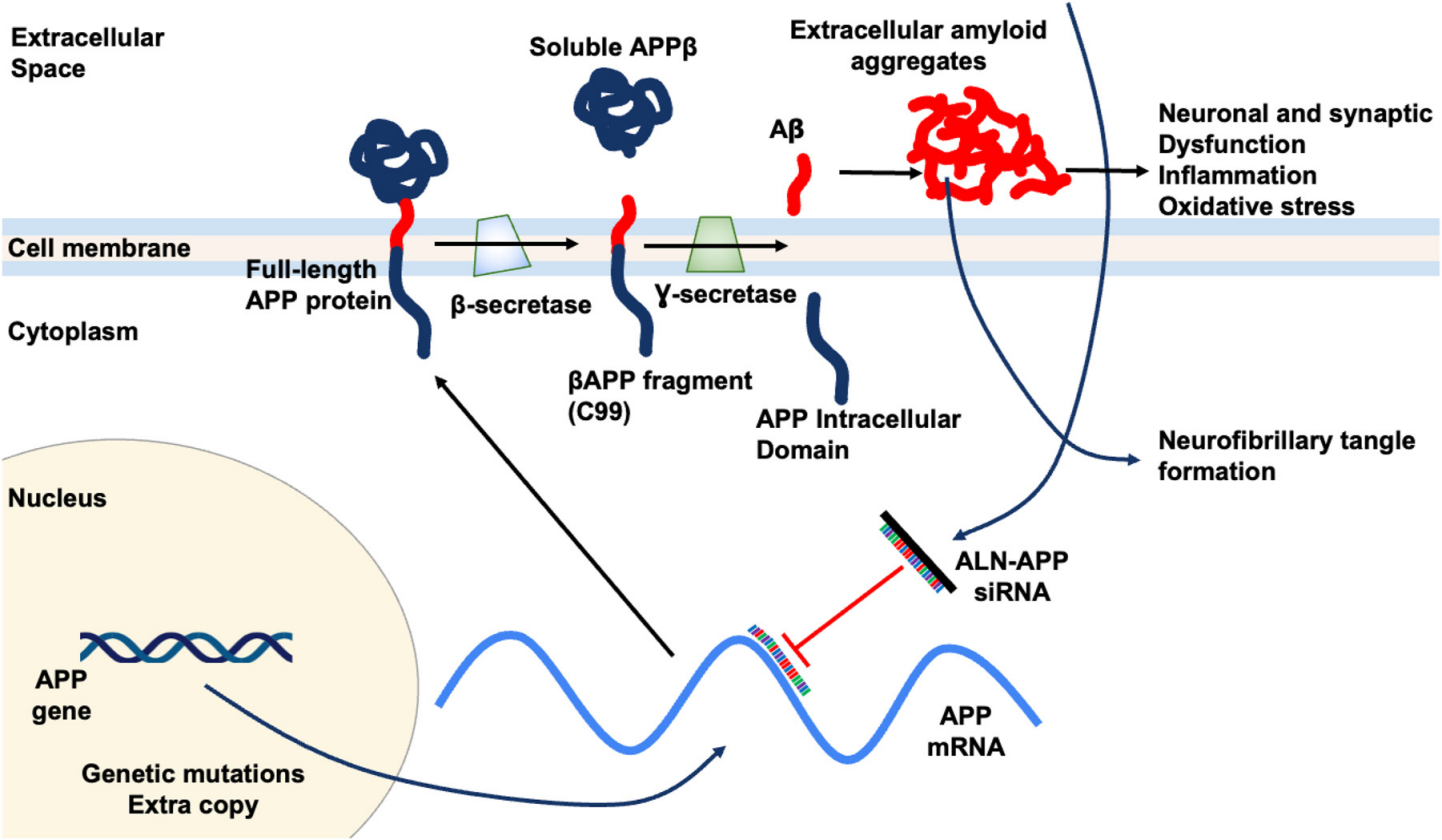
Potential mechanism of action for ALN-APP siRNA. ALN-APP reduces production of APP, the source of downstream Aβ protein species. Reduces substrate for brain amyloid deposition and reduces neuronal dysfunction.

It is unclear precisely which pathogenic pathways cause AD. One of the most important and early pathogenic events in AD is thought to be the abnormal buildup of amyloid β-peptide (Aβ). In AD and other amyloid aggregation diseases such as cerebral amyloid angiopathy (CAA), there is strong evidence of Aβ deposits in the brain [13], 14]. Aβ is a 42-amino acid peptide and derives from the transmembrane glycoprotein amyloid beta precursor protein (APP). The gene for APP is on chromosome 21. In AD, full-length APP is initially translated from APP mRNA before being transported to the lipid bilayer of membranes, where it is cleaved by the protease enzyme β-secretase. Specifically, β-secretase initiates the cleavage of APP to produce the N-terminal soluble APPβ (sAPPβ) and a C-terminal membrane-bound fragment known as C99. Subsequently, another protease enzyme γ-secretase cleaves C99 to release Aβ containing 40 or 42 amino acids, denoted as Aβ 1–40 or Aβ 1–42, respectively, into extracellular space [15]. The peptide Aβ 1–42, which is the main pathogenic peptide, is highly hydrophobic and tends to aggregate into oligomers and fibril (Figure 5). Late pathogenic hallmarks of AD patients include neuroinflammation, neurofibrillary tangles (NFTs) containing hyperphosphorylated tau protein, and plaques made of aggregated forms of the amyloid Aβ [16]. The prevailing theory for AD pathogenesis suggests that Aβ and tau oligomeric aggregates act as soluble neurotoxic agents, leading to the formation of amyloid plaques and NFTs, respectively. Since tau pathology can occur independently of Aβ, it is still controversial to claim Aβ as the sole trigger of tau toxicity that ultimately leading to neuronal loss. Nonetheless, methods that lower Aβ levels remain actively explored as possible interventions for AD [17]. Recent clinical data have shown that two monoclonal antibody medications targeting Aβ, though not able to reverse the disease, can slow the loss of cognitive abilities in Alzheimer’s disease (AD) patients compared to those who received a placebo [18], 19]. The success in targeting Aβ has prompted the exploration of other therapeutic approaches, such as siRNA drugs.

### Preclinical studies of oligonucleotide therapeutics in AD

Therapeutic strategies involving siRNAs have been widely used in preclinical studies of AD. For example, lentiviral vectors expressing siRNAs targeting β-secretase have been shown to improve behavioral deficits in transgenic animal model of AD [20]. Lentiviral vectors are effective at delivering siRNA to the CNS *in vivo*. However, many of them are derived from HIV-1 and other pathogenic viruses, and their application often involves invasive procedures, raising safety concerns and limiting their potential in clinical therapy. Ongoing advancements in siRNA approaches, including the aforementioned chemical modifications and formulation-based LNP delivery systems, have made it increasingly feasible to target the CNS with siRNA drugs, though some of these treatments still require invasive methods, such as intrathecal (IT) injection. For example, systemic administration of siRNA targeting β-secretase through nanodelivery systems have been shown to cross BBB and ameliorate AD-related neuropathological features in APP/PS1 transgenic mice [21], 22]. Preclinical efficacy of C16-siRNAs has been evaluated in transgenic mouse expressing mutant human APPs and found to be effective in reducing APP mRNA and soluble APPα (sAPPα) protein levels by in the ventral cortex and CSF. Furthermore, combined VP and C16 modifications of siRNA targeting APP have demonstrated up to 70 % APP knockdown in the spinal cord and up to 80 % in the brain of non-human primates [5]. Remarkably, these efficiencies were achieved through a single dose of siRNA administered via the IT route. Significant reduction in sAPPα and sAPPβ protein biomarkers were observed, with the strongest effects seen one-week post-dose. APP silencing was sustained at over 75 % APP for approximately 2.5 months, 50 % at 4.5 months, and approaching full recovery at 9 months in most animals, suggesting C16-siRNAs is potent and durable against CNS targets [5]. In addition to Aβ, aggregated, hyperphosphorylated tau is another key contributor to neurodegeneration in AD and other tauopathies, making it another major focus of research for oligonucleotide therapeutics. Tau protein is encoded by the microtubule-associated protein tau (MAPT) gene and is a microtubule-associated protein primarily expressed in neurons. In the PS19 mouse model, which carries the human tau P301S mutation, the use of MAPT ASOs led to reduced tau deposition and seeding capability, mitigated neuronal loss, improved overall survival, and reversed tau pathology [23]. Other factors contributing to neurodegeneration in AD, such as ApoE, are also being actively explored as potential drug targets for oligonucleotide therapeutics in AD [24]. Together, these novel oligonucleotide therapeutics target critical genes involved in AD pathophysiology, potentially mitigating the pathological effect and key disease mechanisms in AD. They have shown promise in improving disease-associated phenotypes in preclinical animal models, offering hope for treating for AD and other tauopathies.

### Preclinical studies of oligonucleotide therapeutics in ALS

In addition to AD treatment, oligonucleotide therapeutics have also been used to treat amyotrophic lateral sclerosis (ALS). ALS is a progressive neurodegenerative disorder characterized by the death of motor neurons, leading to the atrophy of the muscles they innervate. While it is not entirely clear why neurons die in ALS, many genes involved in protein degradation, the cytoskeleton, and RNA processing have been implicated in the disease. These genes invariably lead to neuronal inclusions of abnormal protein aggregation. Among these genes, superoxide dismutase 1 (SOD1) stands out as a promising therapeutic target. Mutations in the SOD1 protein can form intracellular aggregates due to protein misfolding, which is a hallmark of ALS pathology. Mutations in superoxide dismutase 1 (SOD1) are responsible for 20 % of familial ALS. Therefore, therapeutic approach aiming to decrease SOD1 mRNA and protein is predicted to provide clinical benefit. SOD1^G93A^ transgenic mice model, which overexpresses the mutant SOD1 gene, develops adult-onset neurodegeneration of spinal motor neurons and progressive motor deficits that ultimately lead to paralysis [25]. This model has become a primary tool for testing novel therapeutic interventions, including oligonucleotide-based treatments. McCampbell et al. screened over 2,000 ASOs targeting the human SOD1 gene and identified those with excellent *in vivo* efficacies. Administration of the selected ASOs via CSF resulted in widespread distribution throughout the brain and spinal cord. In SOD1^G93A^ mice, Human SOD1 mRNA was decreased in the spinal cord decreased in a dose dependent manner, with up to 75 % reduction of SOD1 following a single injection. The duration of action lasted nearly 10 weeks, with sustained SOD1 mRNA reduction after a single injection in SOD1^G93A^ rats [26]. These preclinical results provided the foundation for advancing new SOD1 ASOs such as tofersen to human clinical trial.

### Clinical studies of siRNA drugs in Alzheimer’s disease

The siRNA drugs are an active area of research for AD. Particularly, early-onset alzheimer disease (EOAD) is a subset of AD caused by genetic changes that affect the proteolysis and expression of APP. This genetic etiology makes EOAD an appealing target for new therapeutics aiming at reducing Aβ production and accumulation [27]. ALN-APP is an intrathecally administered, investigational RNAi therapeutic targeting APP, in development for the treatment of AD and CAA. To increase cellular absorption in the central nervous system, ALN-APP is conjugated to 2′-O-hexadecyl (C16), integrating into the RISC that binds to and reduces APP mRNA. By decreasing APP production, brain amyloid deposition is reduced, potentially facilitating natural clearance. Additionally, by reducing intracellular Aβ, neuronal and synaptic dysfunction, as well as neurofibrillary tangle formation, may decrease. Mechanism of action for ALN-APP is illustrated in Figure 5.

ALN-APP has recently been evaluated in a Phase I clinical trial. It is a randomized, placebo-controlled, single-ascending dose study, consisting of 3 cohorts: 25, 75, and 50 mg [28]. Pooled adverse events (AE), ALN_APP and placebo (PBO), and patient years (PY) are summarized in Table 1. All AEs were mild or moderate in severity. No deaths, SUSARs (suspected unexpected serious adverse reaction), or treatment or study discontinuations occurred. One individual in the 50 mg or placebo cohort had two mild AEs that were deemed drug-related by the investigator and included post-LP (lumbar puncture) headache and post-LP nausea, both of which resolved on the same day. Therefore, this Phase I study shows ALN-APP is well tolerated.

**Table 1:** Pooled adverse event (AE) summary for cohorts 1–3.

N (%)	ALN-APP 25 mg or PBO (N=6, PY=4.1)	ALN-APP 50 mg or PBO (N=8, PY=2.8)	ALN-APP 75 mg or PBO (N=6, PY=3.6)
At least one mild AE	5 (83.3)	6 (75.0)	4 (66.7)
At least one moderate AE	4 (66.7)	4 (50.0)	3 (50.0)
At least one serious AE	0	0	0
Death	0	0	0
At least one AE related to LP	4 (66.7)	6 (75.0)	2 (33.3)

Available clinical data from the Phase I study suggest that ALN-APP generally well tolerated, with all AEs mild or moderate in severity, and is effective in reducing APP products. Lumbar puncture was performed in these cohorts to measure sAPPα and sAPPβ, two soluble peptides generated by α-secretase and β-secretase cleavages downstream of APP. Peak mean (±SEM) reduction in sAPPα was 69 % (±9.6) for the 75 mg dose occurring at Month 2, with a maximum reduction of 84 % observed. Reduction in sAPPα was sustained, with a 56 % (±7.5) mean reduction 6 months after a single 75 mg dose. Similarly, peak mean (±SEM) reduction in sAPPβ was 82 % (±6.3) for the 75 mg dose occurring at Month 2, with a maximum reduction of 90 % observed. Reduction in sAPPβ was sustained, with a 65 % (±9.2) mean reduction 6 months after a single 75 mg dose. Remarkably, these rapid and sustained reductions in sAPPα and sAPPβ were observed following a single dose of ALN-APP in this Phase I study of patients with EOAD. The consistent and sustained reduction in sAPPβ suggests that ALN-APP is effective in reducing sAPPβ levels, potentially offering protection against AD.

### Clinical studies of ASO drugs in Alzheimer’s disease

Like siRNA, antisense oligonucleotide (ASO) has been utilized as a new therapeutic approach for Alzheimer’s disease [29]. In addition to APP, the pathogenesis of Alzheimer’s disease (AD) is significantly related to tau, and more evidence indicates that reducing tau may reduce this pathology. Recent data suggest that lower tau levels in patients with moderate AD and block MAPT (microtubule-associated protein tau) expression using a tau-targeting antisense oligonucleotide (MAPT_Rx_) may provide therapeutic benefits [29]. MAPT_Rx_ is designed to target MAPT messenger RNA and lower its quantities. An 18-nucleotide length of MAPT pre-mRNA is complementary to MAPT_Rx_, a synthetic oligomer that has undergone chemical modification. By Watson–Crick base pairing, MAPT_Rx_ binds to intron 9 of the MAPT pre-mRNA. As a result of hybridization, endogenous ribonuclease H1 degrades the MAPT mRNA, preventing the tau protein from being translated. Phase 1b trial have been performed with MAPT_Rx_, which features multiple ascending doses (MAD), double blind, placebo control, and randomization assessment of the pharmacokinetics, target engagement, and safety. During the 13-week treatment period, followed by a 23-week post-treatment period, four ascending dose cohorts of 46 patients were enrolled consecutively and randomly assigned 3:1 to receive intrathecal bolus doses, at 10, 30, and 60 mg, of MAPT_Rx_ or placebo every 4 or 12 weeks. CSF samples were obtained before the administration of study drug on days 1, 29, 57 and 85 for cohort A (10 mg MAPT_Rx_ or placebo monthly), cohort B (30 mg MAPT_Rx_ or placebo monthly) and cohort C (60 mg MAPT_Rx_ or placebo monthly) and on days 1 and 85 for cohort D (115 mg MAPT_Rx_ or placebo quarterly). Overall, forty-six patients enrolled in the trial, of whom 34 were randomized to MAPTRx and 12 to placebo. Adverse events were reported in 94 % of MAPT_Rx_-treated patients and 75 % of placebo-treated patients [29]; all were mild or moderate. No serious adverse events were reported in MAPT_Rx_-treated patients.

At 24 weeks after the final treatment, the CSF total-tau (t-tau) concentration in the 60 mg (four doses) and 115 mg (two doses) MAPT_Rx_ groups showed a dose-dependent decrease with a mean reduction of more than 50 % from baseline. Twenty-four weeks after the last dose, MAPT_Rx_ treatment caused a 50 % mean reduction from baseline in CSF t-tau and p-tau181 concentrations, which decreased in a dose- and time-dependent manner [29]. The optimal dosage and frequency for further clinical trials will depend on the further characterization of the MAPT_Rx_ PK and PD results from the LTE. In a clinical trial, it is not feasible to measure the decrease of tau protein or MAPT mRNA in cortical tissue directly; nevertheless, CSF protein assays can be used to estimate PD activity. Given that tau is a long-lasting protein in the CNS, CSF tau will serve as a lagging indicator in this study of the decrease in MAPT mRNA and the production of newly synthesized tau in the CNS. The predicted concentrations of MAPT_Rx_ in brain tissue, across all dose levels examined in this investigation, are adequate to yield a reduction of >50 % in the formation of tau in the cerebral cortex. It is therefore not surprising that the CSF tau lowering trajectory at all dosages evaluated in this investigation showed a similar trajectory. This trajectory likely represents strong reductions in new tau synthesis at all doses with a rate-limiting requirement of the removal of existing tau protein.

MAPT_Rx_ is the first ASO treatment evaluated in a clinical study of patients with AD. The significant dose-dependent and sustained decreases in CSF t-tau concentrations shown in the first in human study’s results suggest that MAPT_Rx_ engaged its target, and its safety profile was deemed acceptable for individuals with moderate AD. The treatment of tofersen did not result in statistically significant improvements on clinical endpoints in a Phase III study involving patients with SOD1 amyotrophic lateral sclerosis; however, tofersen was found to be beneficial across clinical outcome measures of respiratory function, muscle strength, and quality of life during the 28-week treatment period, and these improvements continued during the open-label extension, including improvements on clinical endpoints at week 52. Importantly, there was a noticeable reduction in plasma NfL chains and CSF SOD1 protein, which are indicators of neurodegeneration and axonal damage. The use of ASOs to treat neurodegenerative diseases is still in its infancy, but ASO development and clinical trial designs for various neurodegenerative diseases will be improved from the lesson learned from recent research on dose level and frequency as well as trial design (treatment duration, sample size, and patient selection).

### Clinical studies of ASO drugs in amyotrophic lateral sclerosis

Therapeutic approaches aiming to reduce SOD1 are a contested area of ALS drug development. Recently, the reduction of SOD1 protein synthesis by tofersen via intrathecally administration was studied in patients with ALS associated with mutations in SOD1 (SOD1 ALS) [30]. A total of 108 patients with 42 distinct SOD1 mutations were included in this Phase III clinical trial, known as the VALOR trial, 72 of them were assigned to receive tofersen, and the remaining 36 received a placebo. The faster-progression subgroup, comprising 60 participants, was the subject of the primary analysis. Ninety five participants (88 %) from the VALOR trial were included in the open-label extension. Over the course of 24 weeks, randomized patients with SOD1 ALS in a 2:1 ratio were assigned to receive eight doses of tofersen (100 mg) or a placebo.

To evaluate the efficacy of tofersen, the primary endpoint was defined as the change from baseline to week 28 in the total score on the ALS Functional Rating Scale-Revised (ALSFRS-R; range, 0 to 48, with higher scores indicating better function) among participants predicted to have faster progressing disease. Secondary endpoints included changes in the total concentration of SOD1 protein in cerebrospinal fluid (CSF), in the concentration of neurofilament light chains in plasma, in slow vital capacity, and in handheld dynamometry in 16 muscles. The results of the early-start group (trial participants who began tofersen at trial entry) were compared with those of the delayed-start cohort (trial participants who switched from placebo to the medicine at week 28) using a combined analysis of the trial’s randomized component and its open-label extension at 52 weeks. Of the subjects, 72 were given tofersen (39 were predicted to progress more quickly), while 36 were given a placebo (21 were predicted to progress more quickly). Compared to placebo, tofersen caused a larger decrease in the amounts of neurofilament light chains in plasma and SOD1 in CSF. The ALSFRS-R score changed to −6.18 with tofersen and −8.14 with placebo in the faster-progression subgroup (main analysis) at week 28 (difference, 1.2 points; 95 % confidence interval [CI], −3.2 to 5.5; p=0.97).

Ninety-five individuals (88 %) participated in the open label extension. At 52 weeks, there was a difference of 3.5 points (95 % CI, 0.4 to 6.7) in the ALSFRS-R score between the early-start group and the delayed-start cohort (difference, 6.0); non-multiplicity-adjusted differences favoring early-start tofersen were observed for other endpoints. Adverse events associated with lumbar punctures were common. Seven percent of tofersen recipients experienced neurologic serious adverse events. Tofersen decreased SOD1 in CSF and neurofilament light chain concentrations in plasma throughout a 28-week period in patients with SOD1 ALS. Furthermore, there were some trends toward clinical benefit by tofersen that were not statistically significant. However, it did not meet its primary endpoint of slowing the rate of disease progression. The potential effects of earlier as compared with delayed initiation of tofersen are being further evaluated in the extension phase, which showed that earlier initiation of tofersen has clinical benefits in ALS patients. Tofersen is currently being studied in the Phase III ATLAS study, which will evaluate whether it can delay clinical onset when initiated in presymptomatic individuals with a SOD1 genetic mutation and biomarker evidence of disease activity. Based on these available clinical data, and the reduction in plasma NFL concentration provides clinical benefits in tofersen-treated patients with SOD1-ALS, tofersen has been approved by FDA in April 2023.

In summary, oligonucleotide drugs are emerging with several advantages over traditional medicine. First, oligonucleotide drugs have broader therapeutic applications that are limited by the druggability of proteins. They can be designed to target any gene of interest, requiring only the sequence information of the target mRNA. Second, oligonucleotide drugs are relatively safe, requiring only small dosages and low frequency of administration due to its specificity. RNAi and silencing are part of natural biological processes. Therefore, oligonucleotide drugs can be biodegraded, have low cytotoxicity and immunogenicity, and intervene at the mRNA level without affecting the genome. Third, oligonucleotide drugs are less likely to develop drug resistance. Small nucleic acid drugs directly regulate the expression of upstream genes, making them relatively less prone to drug resistance. Fourth, the *in vivo* half-life of small molecule drugs is in the range of hours while antibody drugs are in the range of days/weeks. The siRNA drugs *in vivo* half-life is in the range of months. Further improvements are also required for oligonucleotide drugs to become effective and standard therapeutic medications.

## Conclusions

Oligonucleotide drugs are efficacious, safe, and long acting. The use of oligonucleotides in medicine has overcome several challenges to become one of the most active fields for the creation of new medicines. These challenges include the chemical modifications of RNAi molecules and the development of delivery systems. Naked RNA molecules are not stable and must be chemically modified so that the molecule can be stable enough to survive to the target organs. To effectively deliver RNAi drugs to the target organs, lipid nanoparticle and siRNA conjugate are the two successfully used technologies. Multiple siRNA commercial medications for the hepatic delivery of oligonucleotides have effectively exploited liver-targeted N-acetylgalactosamine (GalNAc) ligand. For extrahepatic delivery, lipid nanoparticles, local delivery, and 2′-O-hexadecyl (C16) are used. A commercial product of siRNA incorporated into lipid nanoparticle was approved by FDA. RNAi drugs have been used in different disease areas including CNS. Preliminary data from the clinical trials of RNAi investigational products including ASO and siRNA show promising results as future medicines to treat neurodegenerative diseases such as AD and ALS.
